# Identifying Critical Errors: Addressing Inhaler Technique in the Context of Asthma Management

**DOI:** 10.1007/s41030-018-0051-0

**Published:** 2018-06-01

**Authors:** Sinthia Z. Bosnic-Anticevich, Biljana Cvetkovski, Elizabeth A. Azzi, Pamela Srour, Rachel Tan, Vicky Kritikos

**Affiliations:** 1 Quality Use of Respiratory Medicines Group, Woolcock Institute, Sydney Medical School, University of Sydney, Sydney, Australia; 2 Woolcock Emphysema Centre, University of Sydney, Sydney, Australia; 3 Sydney Local Health District, Sydney, Australia; 4 Department of Respiratory Medicine, Royal Prince Alfred Hospital, Sydney, Australia

**Keywords:** Asthma control, Critical errors, Health behavior, Health care practitioners, Inhaler devices, Maintenance, Mastery, Skills

## Abstract

Medication use has always played a highly significant role in the overall management of asthma, with appropriate use being linked to good asthma control. However, while patients with asthma enjoy the ‘luxury’ of having medications delivered directly to the lungs via inhaler devices, with that comes the additional challenge of ensuring that inhaler devices are used correctly. Research and practice provides evidence to the challenges associated with inhaler use and the particular steps that patients perform incorrectly. While this problem is well documented, acknowledged and reported, little has changed in 40 years, and the proportion of patients using inhaler devices remains unacceptably high. This review focuses on aspects specific to the errors that patient’s make, the significance of these errors, and the important considerations for health care practitioners in supporting patients in correctly using their inhalers. This review highlights the complexities associated with patient’s making inhaler technique errors and highlights the opportunities that lie in future technological developments of inhaler devices. Now more than ever, in the era of precision medicine, it is important that we address inhaler technique use once and for all.

## The Importance of Medication Use in our Understanding of Respiratory Disease

After decades of research, practice, and perceived knowledge, we find ourselves at cross-roads in our understanding and management of airway diseases, such as asthma [1]. While asthma is an obstructive airways disease, which to our current knowledge can neither be prevented nor cured, it is a disease that fluctuates in severity and ‘control’, is exacerbated by varying triggers, and its course over an individual’s lifetime is essentially unpredictable [1]. While the conversation about asthma and airways disease more broadly is not the focus of this review, it becomes particularly relevant in our consideration of the use of medications. It is critical, now more than ever, that we understand the role of medication use, as it can provide us with further understanding about respiratory disease more broadly [2]. Given that asthma management is reliant on the use of medications [3], ensuring that patients use their medications correctly has become more than just a practical matter; it is a complex and critical issue. Correct medication use has become a fundamental step in our exploration and understanding of disease traits [1, 2]: with careful and more precise evaluation of patient’s response to treatment becoming integral to our understanding of disease endotypes and our evolution towards precision medicine [1, 2]. For this reason, if for no other, it is now more important than ever that we address inhaler use, especially our knowledge of critical errors, which can ‘cloud’ our understanding of treatment success. This article is based on previously conducted studies and does not contain any studies with human participants or animals performed by any of the authors.

## Inhaler Technique and Asthma Control

When it comes to inhaler use, it is accepted that we have a long-standing problem, i.e., a high proportion of patients using inhalers do so incorrectly [4]. Research indicates that the magnitude of this problem is huge with anywhere up to 90% of people with asthma (depending on device and patient population) not using their inhalers correctly, i.e., demonstrating incorrect inhaler technique [5–11]. Poor inhaler technique is associated with suboptimal asthma control, disease instability, and increased hospital visits [5, 7–9, 12, 13]. Its burden extends directly onto health care costs [14], with one-quarter of the costs associated with inhaler use being associated with poor inhaler technique [14].

While not being the sole contributor to poor asthma control [5, 15], the importance and relevance of inhaler technique is further evidenced in the impact of inhaler technique improvement on asthma outcomes. Studies in a range of clinical settings from tertiary care, outpatient clinics, general medical practice, and pharmacies [16–18], that improvement in inhaler technique is associated with improved asthma control [19–22] and asthma-related quality of life [23, 24].

## Inhaler Use in Practice: the Importance of Specific Steps and Errors

In recognizing the impact of inhaler technique on disease control, there are several important details to consider about the specific steps required to use an inhaler correctly.

Firstly, there are many different inhalers available, each requiring a set of specific steps for correct use [25–27]. Patients are required to master the skills specific to their inhaler and while evidence suggests that there may be some carry-over skills between inhalers [28], unless patients are educated on the use of different inhalers, disease outcomes are compromised [29].

Secondly, for each type of inhaler, some steps are more likely to be completed erroneously than others. Sanchis et al. conducted a comprehensive review of the literature, summarizing the frequency with which particular errors were made by patients using difference devices [30]. A summary of the data evidence by Sanchis is included in Table 1 and indicates a large variability between the proportions of subjects making errors across different studies. For example, for the pressurized metered dose inhaler (pMDI), two of the most commonly made errors across the studies were failing to fire the inhaler while breathing in slowly and failing to breath hold after completion of dosing. The proportion of subjects making the former error ranged from 24 to 77%, while the proportion of subjects making the latter error ranged from 10 to 68% across the different studies [30]. For the dry powder inhalers (DPIs), similar variability with overall failure to breathe out and away from the inhaler and failing to breath hold being the most common errors across the different DPI devices. For the DPIs, some errors were more specific to certain devices, e.g., over 30% of subjects were reported to make an error relating to inhaler position for the Turbuhaler^®^ and Rotahaler^®^, and less than 10% of subjects making this same error for the other DPIs [31]. The most common errors (i.e., errors reported to be made by at least 50% of the study population in at least one study) for the pMDI can be generalized and include: failing to breath out prior to inhalation, poor coordination of inhaling slowly and inhaler firing, failing to shake the inhaler, and failing to breath hold following dose delivery [32]. For the DPIs, generalizing the most common errors (i.e., errors reported to be made by at least 50% of the study population in at least one study) is more difficult across the devices and include: failing to breathe out and away from the inhaler prior to inhalation, failing to inhaler forcefully and deeply and breath holding [30].

**Table 1:** Proportion of participants in studies from 2000 to 2013 who make specific errors Modified from Sanchis et al. [30]

Step	pMDI		Dry powder inhalers									
			Turbuhaler		Disckus/Accuhaler		Rotahaler		Diskhaler/rotadisk		Aerolizer/cyclohaer	
	Min	Max	Min	Max	Min	Max	Min	Max	Min	Max	Min	Max
Remove the cap	0%	5%										
Shake the inhaler	7%	57%										
Inhaler positioning	0%	10%	7%	31%	7%	7%	3%	37%	15%	15%	0%	9%
Priming			0%	33%	3%	3%	0%	3%	2%	4%	1%	10%
Breathe out (pMDI) Breath out and away (DPI)	30%	66%	10%	65%	6%	30%	9%	66%	0%	44%	7%	40%
Place inhaler between lips/mouthpiece between lips	6%	16%	0%	28%	2%	2%	4%	29%	4%	15%	0%	1%
Forceful, deep inspiration			2%	55%	–	–	1%	10%	2%	37%	0%	0%
Fire inhaler while breathing in slowly	10%	68%										
Continue to inhale	26%	58%										
Breath holding (5–10 s)	24%	77%	8%	68%	26%	26%	34%	54%	2%	37%	28%	30%

Thirdly, the most common errors are not necessarily the most important errors. Historically, researchers have classified errors as ‘critical’ or ‘essential’, based on an assumption (that if they are not performed correctly, little or no medication reaches the lungs [33]), rather than evidence. This all changed with the research of Price et al. in the CRITIKAL study [7]. The CRITIKAL study explored the use of inhalers by people with asthma with the aim of identifying those errors that are truly ‘critical’, i.e., those errors, which if made, have a negative impact on clinical outcomes (uncontrolled asthma and increased exacerbation rate) [7]. In light of the fact that poor asthma control is a result of multiple factors [2, 15, 34], the CRITIKAL study approached the problem of poor asthma control in a holistic way, with the final analysis being performed and reported for three inhaler devices: the Turbuhaler, the Diskus/Accuhaler, and the pMDI. Price et al. [7] discovered that in a population of people with asthma on fixed-dose combination treatment with inhaled corticosteroids and long-acting beta agonist, several generic and device-specific errors were identified as critical. Taking into account the participant’s age, gender, body mass index, smoking status, rhinitis, and paracetamol use, all of which were associated with uncontrolled asthma and therefore confounders, it was found that the most common error was in fact also a critical error for the Discus/Accuhaler, however, not for the Turbuhaler and the pMDI. The CRITIKAL study showed that the identified ‘critical’ steps also change when patient adherence is taken into account. Table 2 summarizes the results of the CRITIKAL study, highlighting those steps, which are critical to asthma control [7].

**Table 2:** Evidence-based critical errors associated with the use of the Turbuhaler, Diskus/Accuhaler, and pMDI by patients in the CRITIKAL study [7]

Turbuhaler (seven critical errors)	Diskus/Accuhaler (three critical errors)	PMDI (seven critical errors)
**Did not remove cap/slide cover open**	–	Did not remove cap/slide cover open
**Does compromised after preparation because or shaking or tipping**	–	NA
**Insufficient inspiratory effort**	**Insufficient effort**	–
Did not have head tilted such that chin is slightly upward	–	**Did not have head tilted such that chin is slightly upward**
Did not breathe out to empty lungs before inhalation	Did not breathe out to empty lunge before inhalation	Did not breathe out to empty lunge before inhalation
–	–	Exhaled into device before inhalation
Did not seal lips around the mouthpiece	–	Did not seal lips around the mouthpiece
NA	NA	**Actuation did not correspond with inhalation**
No breath hold (or holds breath for < 3 s)	No breath hold (or holds breath for < 3 s)	No breath hold (or holds breath for < 3 s)

^#^Errors in bold identified as critical after adjusting for patient factors (age, sex, body mass index, smoking, rhinitis, and paracetamol use), which were independently associated with uncontrolled asthma and considered potential confounders [7]

Reprinted from [7], with permission from Elsevier

## What do Health Care Practitioners Need to Consider in Addressing Critical Errors?

Clearly and consistently it is recognized that health care practitioners, across the spectrum of health professions, have a responsibility to ensure that patients know how to use their inhalers correctly [11, 17, 26, 35–42], i.e., with minimal critical errors [7]. While at this stage there are no prospective studies establishing the effectiveness of critical error-focused inhaler technique training on disease outcomes, this research will no doubt follow in the future and will provide us with a more precise approach to what has up until now, been a ‘one-size fits all’ approach.

Having established the importance of pursuing the critical error-focused approach, health care practitioners still require guidance with regards to their role in addressing the issue of poor inhaler technique and critical errors.

In 2011, a joint ERS/ISAM taskforce [25] stated the four key principles that health care practitioners (pulmonary specialists) need to know with regards to inhalation therapies: be aware of the devices that are currently available to deliver the prescribed drugs; know the various techniques that are appropriate for each device; be able to evaluate the patient’s inhalation technique to be sure they are using the devices properly; and ensure that the inhalation method is appropriate for each patient. In order to implement the above-noted principles, the realities of inhaler technique and health care practitioners need to be considered.

1. Health care practitioners need to have more than theoretical knowledge about inhalers; they need to be proficient in their use and be able to provide practical advice.

With the availability of inhaler devices ever-increasing, the need for health care practitioners to be proficient and skilled in their use remains a challenge. Unfortunately, research highlights that health care practitioners are not coping. A recent review by Plaza et al. highlights that on average, only 15% of health care practitioners are able to demonstrate correct use of inhalers [43]. This is a major problem if we consider that patients rely on health care practitioners for inhaler technique education. Training health care practitioners in the correct use of inhalers can overcome this lack of knowledge/skills [44] and encouraging them to teach patients can ensure that health care practitioners maintain these skills [45]. There are also a range of common beliefs about inhaler use and health care practitioners need to be able to clarify these for patients [46].

2. Inhaler technique needs to be delivered in an effective format.

Much research has confirmed that patients need to be shown how to use their inhaler, receiving feedback either face-to-face [47] or via video [48], with or without the use of additional tools such as inhaler labels [49] or visual feedback [50]. With this format of educational interventions, we can expect almost all patients to master inhaler technique. Research indicates that this is the case for all devices, even though the time to teach the correct use of different devices ranges from 2 and 8 min depending on the device [47, 51]; pMDIs requiring longer than dry powder inhalers [51]. As part of this process, tools such as the In-CheckDial, Inhalation Manager, Aerosol Inhalation Monitor (AIM), the 2Tone Metered Dose Inhaler Training Device, the SmartMist and multimedia training tools which utilizes video clips have been successfully used to train patients in the use of their inhalers [52, 53]. These tools can be used to support the delivery of inhaler technique education.

3. Inhaler technique needs to be checked and education repeated over time.

It is now well established that having the physical skill to use an inhaler does not guarantee that a patient will continue to use an inhaler correctly over time, with approximately 50% of patients failing to maintain correct inhaler technique over time [54–58]. While there are tools that can be used in addition to physical demonstration to improve this [59], the starting point is a follow-up of inhaler technique over time by the health care practitioner. Recent self-reported data from health care practitioners suggests that just over 50% of prescribers check inhaler technique during a consultation [60]. This clearly needs to increase as does our understanding of how to incorporate inhaler technique review and education into every consultation. It has been suggested that inhaler technique assessment be incorporated into asthma control tools [61]. We are yet to see this happen.

4. Past experience with inhalers matters.

When patients are prescribed a new inhaler, guidelines/reports promote the need to educate the patient, with no distinction regarding first time inhaler users versus patients who have switched from another device. Research indicates that patients who are switched from one device to another are more likely to have poor inhaler technique and disease control if they are not counseled on the switch [62]. Further to this, while not yet tested in patients, recent research exploring the ease of learning how to use new devices (dry powder inhalers) by health care professional students suggests that once trained in the use one inhaler device correctly, there is a ‘learning effect’ for subsequent devices [28], i.e., using the second device may be intuitively easier, as a result of past training. Based on this research, we can hypothesize that learning how to use a second inhaler is only easier when the inhalers are similar; where the devices require significantly different technique, it may make the learning of the second device more difficult. This hypothesis is yet to be tested but certainly needs to be considered by health care practitioners.

5. Patient self-awareness of inhaler technique is poor and communication between the patient and health care practitioner is important.

Patients are often very poor at perceiving issues with their asthma management [6, 63]. This extends to their perception and self-awareness of their ability to use their inhalers, with patients often believing that their inhaler is easy to use [64, 65]. Therefore, health care practitioners cannot rely on patients to self-report issues with inhaler use but need to be proactive and find a way to engage the patient, even when they may not believe they require assistance. In achieving this, it is important to draw on the asthma education literature more broadly. A recent review of asthma education determined that if asthma education is to result in long-term health outcomes, an approach to asthma education which is delivered routinely and keeps the shared patient and health care professional goals in mind, is required [66]. This is supported by research identifying the importance of communication in enhancing asthma patient’s experiences, fostering engagement, and encouraging the patient to follow through their treatment plans [40, 60, 67, 68]. Further to that, a literacy-sensitive, culturally and linguistically appropriate approach should also be incorporated [69–73]. Despite the fact that correct inhaler technique can be taught within 2 and 8 min (depending on the device), the health care practitioner may need to invest additional time with the patient over time, providing sustained experience/multiple visits to change patient’s perceptions of their asthma severity, beliefs, health behavior, and enhanced self-efficacy [74]. As part of this process, perhaps tools like the In-CheckDial, Inhalation Manager, Aerosol Inhalation Monitor (AIM) [52, 53], which are often used to evaluate patients’ breathing maneuvers as they relate to the use of different inhalers, could be used to engage patients around their inhaler technique.

6. Eliminating critical errors and maintaining correct inhaler technique over time needs to be reconceptualized.

Over the last 40+ years, training patients on the use of their inhalers has been an effort in training patients how to develop the right physical skills to do so. This is reflected in the content of checklists, in the description of the processes, and on the method of evaluation, i.e., following a structured, itemized list of physical steps. However, there is mounting evidence that ensuring that patients use their inhaler correctly over time is a more complex process.

While learning how to use an inhaler correctly can be referred to as a ‘physical skill’, it is maintaining that skill over time that is of critical importance and where the evidence points to a more complex concept.

It has been identified that inhaler technique maintenance is related to patient psychosocial factors, such as motivation [55] and potentially personality traits more intrinsically linked to overall medication taking [75]. That is, there is substantial evidence that inhaler technique and health behaviors such as poor adherence co-exist [5, 7, 12, 18, 34, 57, 76–80] and emerging evidence that they may be related. In the past, poor inhaler technique has been referred to as a type of unintentional non-adherence [57, 81–83], however, more recent evidence strengthens our understanding of this relationship [84, 85]. It is now known that inhaler technique maintenance is related to patient psychosocial factors, such as motivation [55], which in turn is embedded within the patients’ perceptions of the threat posed by their asthma, their perceived confidence in the strategies suggested for managing their asthma, and their confidence in carrying out those strategies [86]. These findings are consistent with those of Jahedi and colleagues [64], which highlight patient perceptions of their asthma and its management, to be linked to inhaler technique. The most recent evidence advances our understanding of the relationship between adherence and inhaler technique even further, showing that patient adherence can be predictive of future inhaler technique maintenance [56]. Therefore, perhaps while mastering inhaler technique may be a skill, maintaining correct technique over time might be better considered and addressed as a health behavior.

## How can Future Treatments/Inhaler Designs Best Address Inhaler Technique Errors

Consideration of how inhaler technique errors might be addressed in the future brings up an interesting discussion. It could be postulated that, with the emergence of biological agents for the treatment of asthma, none of which are administered via inhalation (all of which are currently only indicated for severe asthma), there will no longer be a need to use inhaler devices, providing a permanent solution to poor inhaler technique. However, the more realistic short-term future is the evolution of inhalers which are developed with ease of use in mind and the development of inhalers with e-connectivity aimed at improving adherence and inhaler technique [87].

Currently, there is limited data exploring the impact of the new ‘easier to use’ devices on inhaler technique and clinical outcomes in real life. There is a trend towards few inhaler technique errors [88, 89] and patient satisfaction favoring the newer devices, however, real-life, long-term data are not as yet available, and there is a call for well-designed studies in the future [90]. Until that time, training patients in all the steps required to use these newer inhaler devices is recommended [3].

What is particularly exciting is the possibilities enabled by inhaler devices with e-connectivity [41, 91], i.e., devices which include microprocessors allowing for real-time adherence (by date and time stamping actual use) and inhaler technique monitoring.

Emerging into research and practice are a range of built-in or add-on devices, enabling the monitoring of adherence (through date and time stamping dose delivery) [92–94] and the monitoring of inhaler technique with and without feedback, in real time [92, 95, 96]. For example, the SmartTrack device, which can be attached to a pMDI, delivers reminders for dose administration through customized ringtones [97, 98]. While in addition to monitoring adherence, the INCA device monitors Diskus/Accuhaler inhaler technique using an acoustic-based method [95, 99–102]. Further to monitoring and recording, devices with e-connectivity are being developed enabling patients to receive real-time feedback about their inhaler technique every time they use their inhaler. In a recent proof-of-concept study conducted in children with asthma, the use of smart inhalers resulted in the improvement of inhaler technique which continued even after the feedback from the inhaler was withdrawn [103]. In fact, even though the future capability of e-connectivity and mHealth is still to be realized, there is such enthusiasm for it, its role in research and clinical practice [94], that it has been proposed that all inhalers include this monitoring capability [104]. Where this will take us in the future is as yet unknown, but most certainly it will impact the way inhaler devices are used and the way in which health care practitioners engage with their patients around their use.

## Conclusions

Correct inhaler technique is important in achieving good clinical outcomes for patients with asthma and for several of the commonly used devices; we know exactly which steps are critical. Health care practitioners need to make sure that they know how to use each inhaler they prescribe and commit to training the patient in the correct use, utilizing a placebo inhaler. This means that health care practitioners need to ensure that patients are able to master these critical steps, by assessing and regularly re-assessing their patient’s skills and medication taking behavior. Future research needs to determine the critical errors for all inhalers and how to engage patients to ensure that mastery of good inhaler technique skills becomes a long-term health behavior. The emergency of mHealth is set to play an important role in this field of research and practice in the future.
